# Comparative analysis of plastid genomes within the Campanulaceae and phylogenetic implications

**DOI:** 10.1371/journal.pone.0233167

**Published:** 2020-05-14

**Authors:** Chun-Jiao Li, Ruo-Nan Wang, De-Zhu Li

**Affiliations:** 1 Germplasm Bank of Wild Species, Kunming Institute of Botany, Chinese Academy of Sciences, Kunming, Yunnan, China; 2 College of Life Sciences, Northwest University, Xi’an, China; National Cheng Kung University, TAIWAN

## Abstract

The conflicts exist between the phylogeny of Campanulaceae based on nuclear ITS sequence and plastid markers, particularly in the subdivision of Cyanantheae (Campanulaceae). Besides, various and complicated plastid genome structures can be found in species of the Campanulaceae. However, limited availability of genomic information largely hinders the studies of molecular evolution and phylogeny of Campanulaceae. We reported the complete plastid genomes of three Cyanantheae species, compared them to eight published Campanulaceae plastomes, and shed light on a deeper understanding of the applicability of plastomes. We found that there were obvious differences among gene order, GC content, gene compositions and IR junctions of LSC/IRa. Almost all protein-coding genes and amino acid sequences showed obvious codon preferences. We identified 14 genes with highly positively selected sites and branch-site model displayed 96 sites under potentially positive selection on the three lineages of phylogenetic tree. Phylogenetic analyses showed that *Cyananthus* was more closely related to *Codonopsis* compared with *Cyclocodon* and also clearly illustrated the relationship among the Cyanantheae species. We also found six coding regions having high nucleotide divergence value. Hotpot regions were considered to be useful molecular markers for resolving phylogenetic relationships and species authentication of Campanulaceae.

## Introduction

The three closely related families, Campanulaceae, Cyphiaceae, and Lobeliaceae are sometimes treated as subfamilies of the broadly delimited Campanulaceae which consists of more than 2300 species with nearly cosmopolitan distribution [1]. Campanulaceae *sensu stricto* (*s*.*str*.) primarily distributes in the temperate regions and is centered in East Asia, incorporating three groups of the Platycodonoids, Wahlenbergioids, and Campanuloids based on the capsule dehiscent mode and location of carpel and calyx lobes [2]. Later, Hong and Wang combining the data from palynology, external morphology and DNA fragments, established a classification with three tribes for Campanulaceae *s*.*str*., i.e., Cyanantheae, Wahlenbergieae and Campanuleae [3, 4].

Many Cyanantheae species are important traditional medicines, such as *Platycodon grandiflorus* and *Codonopsis pilosula* showing anti-epileptic, anti-oxidative, anti-viral, and anti-inflammatory properties and some species e.g., *Cyananthus incanus* and *Cyananthus formosus* with ornamental values [5–8]. However, less attention has been paid to this group; there are a few taxonomic and phylogenetic studies apart from the research of medicinal value [9, 10]. The Cyanantheae is distinct from other two tribes by colpate or colporate pollen with elongate apertures and a loculicidal capsule or a berry. The subdivision of this group is still controversial since *Codonopsis*, the largest genus among the Cyanantheae is polyphyletic [4, 11]. The controversies mainly exist in the relationship of *Codonopsis* and its allies. *Codonopsis* is mainly distributed in the Himalayas and southwest China. Studing this genus will be helpful to clarify the phylogenetic relationships of Cyanantheae. In the past years, the nuclear ribosomal ITS and several plastid genome regions (such as *atpB*, *matK*, *rbcL*, *petD*) or their combinations had been frequently used in the study of molecular systematics of Cyanantheae [9, 11]. The selected loci failed to provide sufficient systematic information among Cyanantheae species. Some important branches still show the low supported value and are undefined [4, 9, 11, 12]. As a result, it is necessary to seek other methods for rebuilding the classification of Cyanantheae. Whole plastid genome or hyper-variable regions are urgently needed. The broadly definition of this clade comprises *Platycodon*, *Canarina*, *Cyclocodon*, *Echinocodon*, *Codonopsis and Cyananthus* et al. [4]. Except the *Canarina*, other genera are only found in East Asia. Obviously, the species of East Asia play a vital role in analyzing the genome evolution and demonstrating the phylogenetic relationship of Cyanantheae. *Cyclocodon* and *Cyananthus* are noteworthy in the flora of the Himalayas and adjacent areas. Alpine species of *Cyanathus* endemic to the Himalaya-Hengduan Mountains, have been used to study the distributional responses to climate change [13]. For the species of *Cyclocodon*, calyx lobes are stripe or strip-lanceolate and have dentate margin or rarely entire. *Cyananthus* is a distinctive member of Campanulaceae due to the superior calyx and corolla, which illustrate that this genus appears earlier [14]. Plastid genomes of these floras remain not to be elucidated. What’s more, the plastid genome evolution in Cyanantheae is still blank.

In recent years, based on genomic resources, such as complete plastid sequences, there is a good chance to study the genomic evolution and interspecific relationships of organisms [15–18]. Chloroplasts are small organelles inside the cells of plants with the function of providing photosynthetic machinery and producing essential energy. The majority of the plastid genomes of land plants have highly-conserved compositions, with respect to the gene content and gene order [19–22]. Nevertheless, many rearrangements are the rare evolutionary events and often have certain phylogenetic significance [23]. Various plastid genome structures can be found in the Campanulaceae species because of numerous rearrangements [9, 24–26]. However, the research on plastome structures of Campanulaceae has been relatively scarce [24, 27]. Besides, the conflicts still exist between the phylogeny of Campanulaceae based on ITS and based on plastid markers [4, 11]. Until now, there are few studies of constructing Campanulaceae phylogeny based on the plastomes. Therefore, using the plastid genome structures will be helpful to identify the uncertainty phylogenetic relationships and clarify the structural evolution. Plastid markers and genetic information of more complete plastid genomes of Campanulaceae will also further contribute to the conservation strategy and utilization of this family.

Here, we report newly sequenced complete plastid genomes of *Cyananthus flavus*, *Cyclocodon parviflorus*, and *Codonopsis hongii* using next-generation sequencing technology and genomic comparative analysis with other eight published plastome sequences of Campanulaceae download from the NCBI. The main objectives of this study are to (1) assemble and annotate the genome structures of three Cyanantheae species, (2) reveal structural and size variation in the plastomes of Campanulaceae, and trace the evolutionary pattern of IR expansion/contraction, (3) identify divergence hotspots of plastome regions for further evolutionary and systematic study of Campanulaceae and determine signatures of positive selection, and (4) test the applicability of plastid phylogenomics in resolving phylogenetic relationships of Campanulaceae *s*.*str*., especially within the Cyanantheae.

## Materials and methods

### Plant material, DNA extraction, and sequencing

There is no specific permits required for obtaining the healthy and fresh leaves of *Cyananthus flavus*, *Cyclocodon parviflorus*, and *Codonopsis hongii*, since they are not endangered or protected species and were collected from the fields that are not privately owned or protected. The plant materials of *Cyananthus flavus*, *Cyclocodon parviflorus*, and *Codonopsis hongii* were collected at Lijiang City (27°0'24.4"N, 100°10'31.1"E, alt. 3439 m), Cangyuan Wa Autonomous County (23°14'39"N, 98°56'55"E, alt. 946 m), Gongshan Derung and Nu Autonomous County (27°43'44.1"N, 98°21'34.4"E, alt. 1660 m) of Yunnan, China, respectively.

The voucher specimens of three species were deposited at Herbarium of Kunming Institute of Botany, Chinese Academy of Sciences (KUN). The voucher numbers are KUN 1379897 (*Cyananthus flavus*), KUN 1380108 (*Cyclocodon parviflorus*), and GLGS21262 (*Codonopsis hongii*). Total genomic DNA was isolated from silicagel-dried leaves by using a CTAB protocol [28]. The quality and concentration of DNA were evaluated via agarose gel electrophoresis and spectrophotometry (NanoDrop-2000, Thermo Fisher Scientific). We used an ultrasonicator to randomly fragment the extracted genomic DNA into 400-600bp following manufacturer’s manual (Illumina). DNA libraries with 500-bp insert size were constructed by the NEBNext® Ultra™ II DNA Prep Kit for illumina. Sequencing of paired-end 150 bp read lengths was run on Illumina HiSeq X TEN at Plant Germplasm and Genomics Center of Kunming Institute of Botany. The sequencing quantity of all newly sequenced species is more than 1 Gigabyte.

### Plastid genome assembly and annotation

**Fig 1:**
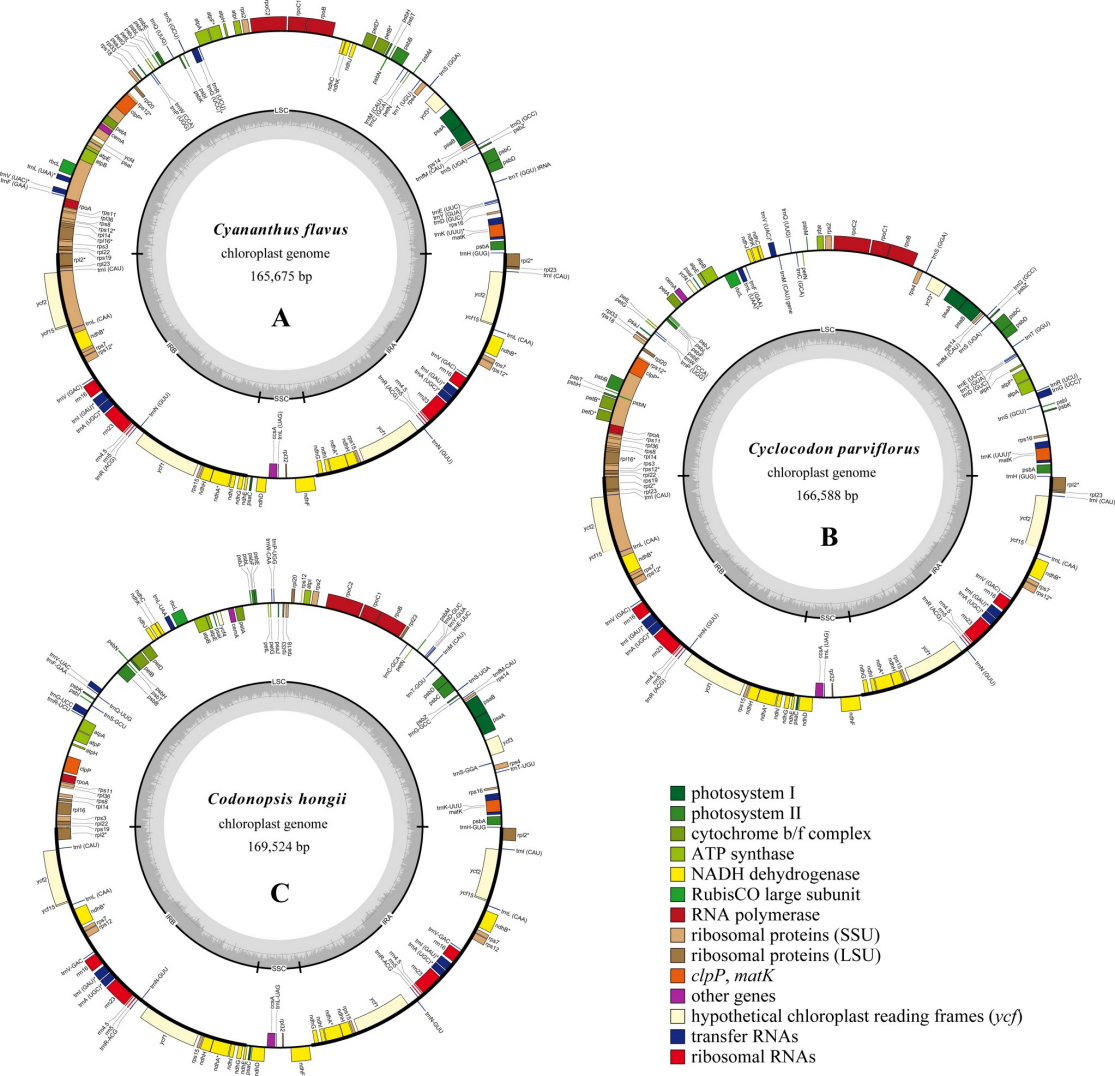
Plastid genomes of *Cyananthus flavus* (A), *Cyclocodon parviflorus* (B), and *Codonopsis hongii* (C). Genes inside the circle are transcribed clockwise, and genes outside the circle are transcribed counter-clockwise. The dark-gray inner circle corresponds to the GC content, and the light-gray represents the AT content.

**Fig 2:**
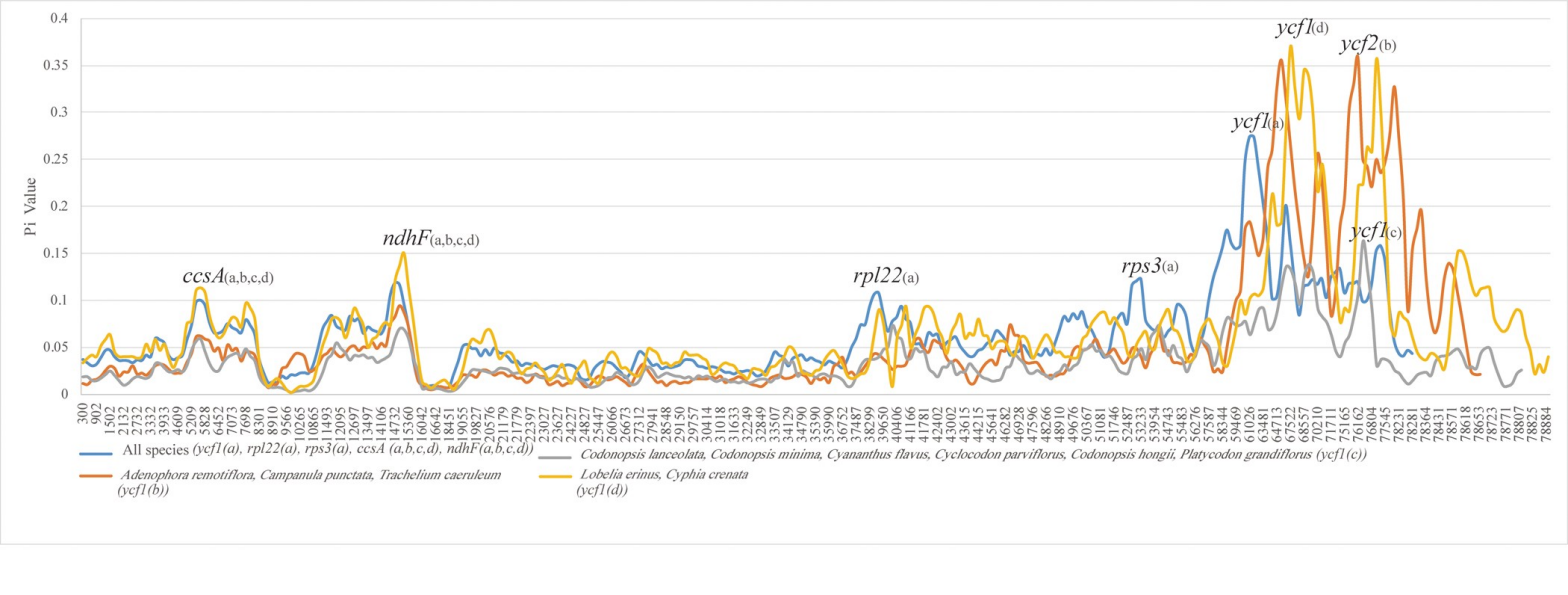
Percentages of variable sites in protein-coding regions. The blue line indicates the comparison of eleven species among the family Campanulaceae; the gray line indicates the comparison of six Cyanantheae species; the orange line indicates the Campanuleae species; the yellow line indicates the out-group. X axis: position of the midpoint of a window. Y axis: nucleotide diversity of each window.

**Fig 3:**
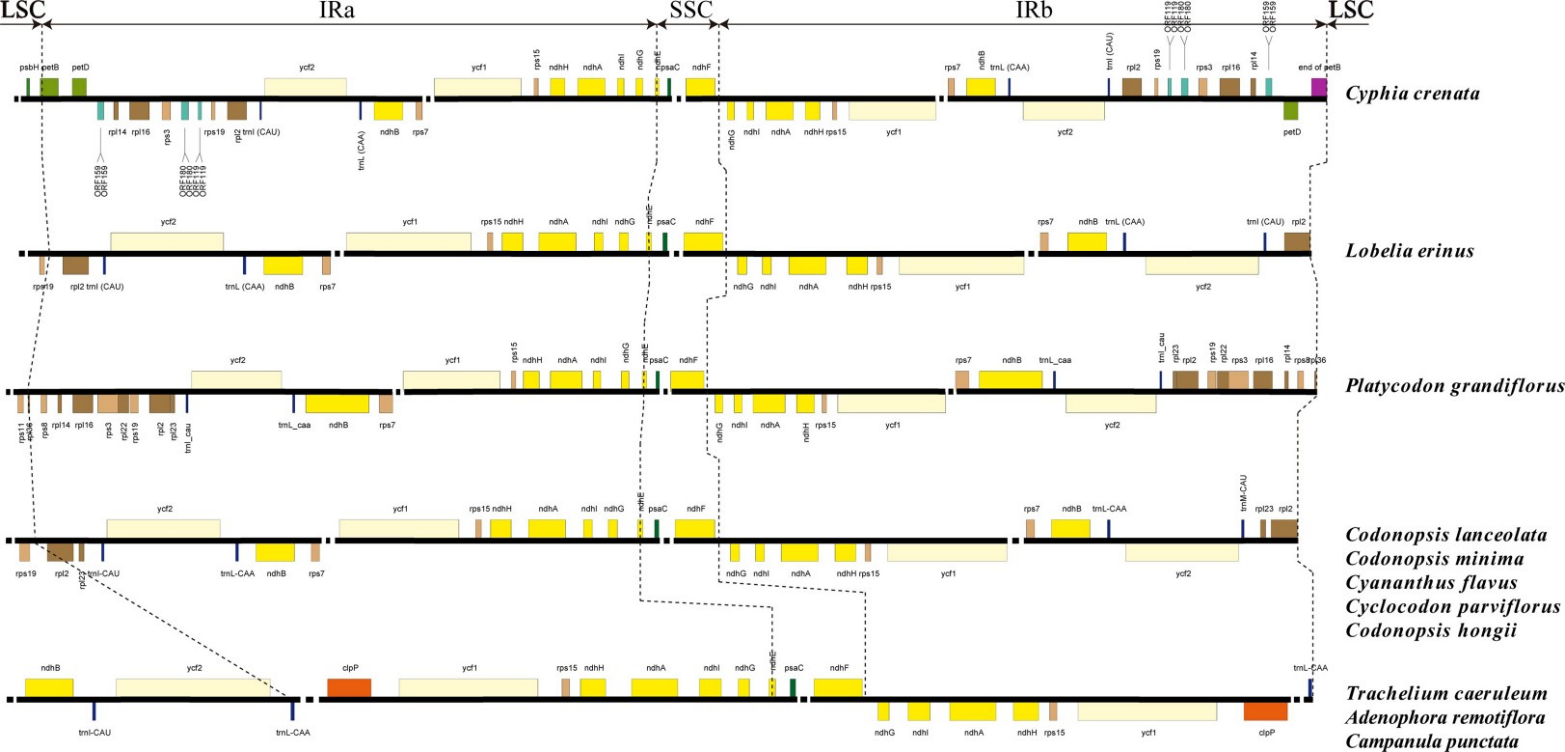
Comparison of the borders between IR and LSC/SSC regions and the gene composition of IR regions.

Complete plastid genome of *Codonopsis lanceolata* (KP889213) as reference, the paired-end reads were filtered and assembled into a complete plastome using GetOrganelle (https://github.com/Kinggerm/GetOrganelle) [29]. The final assembly graph was viewed and checked by Bandage [30] to confirm the paths of the plastomes. In addition, the four junctions between the IR (inverted repeat) regions and LSC (large single copy region)/ SSC (small single copy region) were reconfirmed by PCR and Sanger sequencing. The primers were designed based on the reference genome (*Codonopsis lanceolate* MH018574) through the Primer3 algorithm (http://frodo.wi.mit.edu/primer3/) with the default setting and displayed in the S1 Appendix which also showed the PCR reactions. Sanger sequencing was finished in the BioSune company after purify the them through precipitation with 95% ethanol and 3-sodium acetate. Geneious 8.0.2 [31] was used to align the sanger sequences and assembled genomes for checking any differences. The assembled plastid genome was automatically annotated using PGA [32], then manually adjusted in Geneious. Circular plastid genome maps of *Cyananthus flavus*, *Cyclocodon parviflorus*, and *Codonopsis hongii* (Figs 1, 2 and 3) were drawn using OGDRAW tool [33] with default settings and checked manually. The sequence of plastomes generated in this study was submitted to the NCBI database with the GenBank accession number (Table 1).

**Table 1:** Comparison of plastome features of Campanulaceae species.

Species	*Cyananthus flavus*	*Cyclocodon parviflorus*	*Codonopsis hongii*	*Platycodon grandiflorus*	*Codonopsis lanceolata*	*Codonopsis minima*	*Adenophora remotiflora*	*Campanula punctata*	*Trachelium caeruleum*	*Lobelia erinus*	*Cyphia crenata*
**Accession number**	MT074354	MT074353	MN849357	KX352464	MH018574	KY587457	KP889213	KU198434	EU090187	MF770635	MF770625
**Genome size (bp)**	165675	166588	169524	171818	169447	169321	171724	169341	162321	166019	178956
**LSC length (bp)**	82501	83994	85326	79112	85253	85506	105555	102323	100110	81503	79041
**SSC length (bp)**	8120	8014	7912	7840	8060	8067	11295	7744	7661	7792	8085
**IR length (bp)**	37527	37290	38143	42433	38067	37874	27437	29637	27276	38362	45915
**Coding sequences (bp)**	88242	88587	88296	90144	88761	87332	76245	78771	71508	89784	92511
**Perscent of coding sequences(%)**	53.26	53.18	52.08	52.46	52.38	51.58	44.39	46.52	44.05	54.08	51.69
**Non-coding sequences (bp)**	77433	78001	81228	81674	80686	81989	95479	90570	90813	76235	86445
**Number of genes**	136(25)	135(25)	135(24)	146(32)	138(24)	133(24)	140(21)	134(18)	147(22)	139(25)	150(33)
**Number of protein-coding genes**	89(13)	91(14)	90(13)	95(20)	95(19)	86(12)	82(7)	83(7)	83(7)	89(13)	99(21)
**Number of tRNA genes**	37(7)	36(7)	37(7)	36(7)	38(7)	37(7)	37(5)	36(5)	44(7)	36(7)	36(7)
**Number of rRNA genes**	8(4)	8(4)	8(4)	8(4)	8(4)	8(4)	8(4)	8(4)	8(4)	8(4)	8(4)
**Total GC content (%)**	38.1	37.9	38.2	38.1	38.2	38.3	38.8	38.8	38.3	39.0	36.8
**GC content in LSC(%)**	36.6	36.7	36.9	37.2	36.9	36.9	37.5	37.8	37.1	38.4	35.8
**GC content in SSC (%)**	31.1	31.3	32.0	31.0	32.4	32.4	34.9	32.6	32.2	33.5	31.0
**GC content in IR (%)**	40.4	40.0	40.4	36.9	40.4	40.5	42.0	41.4	41.4	40.2	38.2

### Genome structure analyses and genome comparison

Six plastomes of Campanulaceae *s*.*str*. available in GenBank (Table 1) were included as closely related groups. Among of these, three species are the Campanuleae plants. Additionally, *Lobelia erinus* (Lobelioideae) (MF770635) and *Cyphia crenata* (Cyphioideae) (MF770625) were assigned as the out-group to reconstruct phylogenetic relationships. The whole plastid genomes of eleven species, including the three newly sequenced Cyanantheae species in this study were performed using Mauve [34]. We calculated the ORFs (opening reading frame) >300 bp in the IRa regions of each species in the Geneious. The boundaries between the IR and SSC regions, IR and LSC regions, plus the different contents of IR were compared and analyzed. In total, 76 protein coding genes of all studied species were compiled into a single file and aligned with MAFFT [35] and manually adjusted with Geneious. In addition, the *rpl23* and *infA* genes were excluded from the data matrix, since there being too many losses there. To compare nucleotide diversity (pi) in different groups, we divided the eleven samples into the groups of all species, the Cyanantheae, the Campanuleae, and the out-group. The Pi throughout the coding regions with 200 bp step size and 600 bp window length was determined via the DnaSP version 6 [36] software.

The distribution of codon usage with the relative synonymous codon usage (RSCU) value and the GC content were calculated using the software MEGA 6.0 [37]. RSCU represents the ratio of the observed frequency of a codon to the expected frequency and is a good indicator of codon usage bias [38, 39]. When the RSCU value is less than 1, synonymous codons are used less frequently than expected; otherwise, the value is greater than 1 [40]. The visualization of codon usage in the form of heatmaps of Campanulaceae species and a histogram were conducted with R language with an RSCU value.

### Repeat sequence analyses

REPuter [41] was hired to identify dispersed repeats, including forward (F), reverse (R), palindrome (P), and complement (C) repeats. The repeat sizes were limited to a minimum of 50 bp and the maximum computed repeats were detected less than 100, with a Hamming distance of 3. The IRb of each plastome was removed before the repeat detection, and then the location of repeats in IRb as manually found based on those detected in IRa. We used online Tandem repeats finder (http://tandem.bu.edu/trf/trf.html) to identify tandem repeats sequences with default parameters. Simple sequence repeats (SSR or microsaltellites) in the eleven genomes were detected by A Perl script MISA [42]. Tandem repeats (1–6 nucleotides) were viewed as microsatellites, with the minimal repeat number set to 12, 6, 5, 5, 5 and 5 for mono-, di-, tri-, tetra-, penta-, and hexa- nucleotides, respectively. All of the repeats were manually verified. We also counted the repeat numbers in the regions of LSC, SSC and IRa.

### Positive selection analysis

In order to detect the protein-coding genes under selection within the species of Campanulaceae, we used Muscle (codon) implemented in MEGA to align the each gene. We analyzed all CDS gene regions, except the *rpl23* and *infA*. A Maximum likelihood phylogenetic tree based on CDS regions was constructed using RAxML [43]. The codon substitution models were performed for calculating the non-synonymous (dN) and synonymous (dS) substitution rates, along with their ratios (ω = dN/dS), which were implemented in the Codeml program, PAML3.15 [44]. We used the site-specific model of M0, M1a, M2a, M3, M7, and M8. This model allowed ω ratio to vary among sites with a fixed ω ratio in all branches. M1a (neutral) vs. M2a (positive selection), M7 (β) vs. M8 (β and ω), and M0 (one-ratio) vs. M3 (discrete), were calculated in order to detect positive selection, by comparing the site-specific model [45]. Likelihood ratio test (LRT) of the above comparison was conducted respectively to evaluate the selection strength and the *p*-values of Chi square (x^2^) smaller than 0.05 was thought as significant.

The branch-site model with difference ω among branches (labeled foreground-lineages) of the phylogeny and sites, were also used to test which sites were influenced by the positive selection in the foreground-branch and conducted using the CODEML algorithm [44] executed in EasyCodeML [45, 46]. We took three main lineages of Cyanantheae, Campanuleae and out-group as the foreground branch separately and calculated the positive selection occurred on the aboved branches by using 76 protein-coding genes individually. If the LRT *p*-values were significant (<0.05), Bayes Empirical Bayes (BEB) method [47] was implemented to calculate posterior probabilities for finding sites under positive selection on the three branches [48].

### Phylogenetic analyses

A total of 76 common protein-coding genes shared in the plastomes of Campanulaceae were aligned with MAFFT [33] and were manually adjusted. *Lobelia erinus* and *Cyphia crenata* were selected as the out-group (Table 1). Maximum likelihood (ML) analysis were implemented using RAxML with a bootstrap of 1000 repetitions [42], and the best tree in a single run were found by using the GTR+G model consulted from the RAxML instruction. The jModelTest 2.0 program [49] was used to determine the best-fitting model for dataset based on the Bayesian information criterion (BIC). Regarding Bayesian inference (BI), two independent chains (burinin = 1000) was performed using the program MrBayes v3.2 (Ronquist et al. 2012) at the CIPRES Science Gateway website (http://www.phylo.org/) [50], with the GTR+I+G model determined by jModelTest in the unpartitioned dataset. The Markov chain Monte Carlo (MCMC) analysis was run for 2×1000,000 generations, with trees sampled every 1,000 generations. The first twenty-five per cent of trees calculated were removed as burn-in and the tree of a majority rule consensus would be generated from the remaining trees. The average standard deviation of split frequencies equal to or less than 0.01 would be considered the convergence of the MCMC chains. Figtree v1.4 (http://tree.bio.ed.ac.uk/software/figtree/) was used to visualize and annotate trees.

## Results and discussion

### General features of the plastid genomes

In this study, we first determined the whole plastid genomes of three Cyanantheae species. The mean coverages of *Cyananthus flavus*, *Cyclocodon parviflorus* and *Codonopsis hongii* were 679x, 483x and 1000x, respectively, and the clean reads of the above species were 2,926,584 to 8,710,738. The complete plastid genomes of *Cyananthus flavus*, *Cyclocodon parviflorus*, and *Codonopsis hongii* displayed a typical quadripartite structure and were circular molecular 165,675bp-169,524bp in size (Fig 1 and Table 1). A total of seven protein-coding genes and six tRNA genes contained one intron, whereas three genes (*rps12*, *clpP*, *ycf3*) contained two introns, as shown in Table 2. *Ycf3* gene expression result in stable accumulation of photosystem I complexes [51].

**Table 2:** List of genes present in three newly sequenced plastomes.

Category of genes	Group of gene	Name of gene
**Self-replication**	Ribosomal RNA genes	*rrn16*^(×2)^, *rrn23*^(×2)^, *rrn4*.*5*^(×2)^, *rrn5*^(×2)^
	Transfer RNA genes	trnA-UGC*^, (×2)^, *trnC-GCA*, *trnD-GUC*, *trnE-UUC*, *trnF-GAA*, *trnfM-CAU*, *trnG-GCC*, trnG-UCC*, *trnH-GUG*, *trnI-CAU*, trnI-GAU*^, (×2)^, trnK-UUU*, *trnL-CAA*, trnL-UAA*, *trnM-CAU*, *trnN-GUU*, *trnP-UGG*, *trnQ-UUG*, *trnR-ACG*, *trnR-UCU*, *trnS-GCU*, *trnS-GGA*, *trnS-UGA*, *trnT-GGU*, *trnT-UGU* ^(a,b)^, *trnV-GAC*, trnV-UAC*, *trnW-CCA*, *trnY-GUA*
	Small subunit of ribosome	*rps2*, *rps3*, *rps4*, *rps7*^(×2)^, *rps8*, *rps11*, rps12**^, (×2)^, *rps14*, *rps15*^(×2)^, *rps16*, *rps18*, *rps19*
	Large subunit of ribosome	rpl2*^, (×2)^, *rpl14*, rpl16*, *rpl20*, *rpl22*, *rpl23*^(a,c×2)(d×1)^, *rpl32*, *rpl33*, *rpl36*
	DNA-dependent RNA polymerase	*rpoA*, *rpoB*, *rpoC1*, *rpoC2*
**Genes for photosynthesis**	Subunits of NADH-dehydrogenase	ndhA*^, (×2)^, ndhB*^, (×2)^, *ndhC*, *ndhD*, *ndhE*, *ndhF*, *ndhG* ^(×2)^, *ndhH* ^(×2)^, *ndhI* ^(×2)^, *ndhJ*, *ndhK*
	Subunits of photosystem I	*psaA*, *psaB*, *psaC*, *psaI*, *psaJ*, ycf3**, *ycf4*
	Subunits of photosystem II	*psbA*, *psbB*, *psbC*, *psbD*, *psbE*, *psbF*, *psbH*, *psbI*, *psbJ*, *psbK*, *psbL*, *psbM*, *psbN*, *psbT*, *psbZ*
	Subunits of cytochrome b/f complex	*petA*, petB*, petD*, *petG*, *petL*, *petN*
	Subunits of ATP synthase	*atpA*, *atpB*, *atpE*, atpF*, *atpH*, *atpI*
	Subunits of rubisco	*rbcL*
**Other genes**	Maturase	*matk*
	Protease	clpP**
	Envelope membrane protein	*cemA*
	C-type cytochrome synthesis gene	*ccsA*
**Genes of unknown function**	Conserved open reading frames	*ycf1* ^(×2)^, *ycf2* ^(×2)^

a gene is in *Cyananthus flavus*; c gene is in *Cyclocodon parviflorus*; d gene is in *Codonopsis hongii*;

* gene contains one intron;

** gene contains two introns; (×2) indicates that the number of the repeat unit is 2.

The size of the *Cyphia crenata* plastid genome (178,956bp) was the longest, and that of the *Trachelium caeruleum* plastid genome (162,321bp) was the shortest. Interestingly, the LSC region (79,041bp) of the *Cyphia crenata* was the shortest, while its IR region (45,915bp) and the coding region (92,511bp) were the longest among the studied species, which might be related to the expansion of the border positions between the LSC and IR regions [52, 53]. The length of LSC regions of Campanuleae species was 100,110bp-105,555bp, which were longer than the other species, whereas this group had the shortest IR, with length of 27,276bp-29,637bp, which might be caused by the contraction between the LSC and IR regions. The size of plastid genome was similar among the six species of Cyanantheae (Table 1), apart from *Platycodon grandiflorus* with the longest IR region and shortest LSC and SSC region among the species of this group.

As shown in Table 2, 44.05%-46.52% sequences of plastid were responsible for coding among the Campanuleae species, but more than half sequences being in charge of coding among the other studied species. The GC contents of the LSC and SSC regions in all studied species (except for *P*. *grandiflorus*) were slightly lower than those of the IR regions. The *Lobelia erinus* plastid genome had the highest GC content (39.0%), while the *Cyphia crenata* plastid genome had the lowest GC content (36.38%). For the Campanuleae species, they showed more GC content in the IR region (41.1% or 142.0%). The overall GC content is an significant species indicator [54]. In addition, 80 or 83 protein-coding genes were identified in the Campanuleae species, with 7 genes in the IR regions. 86–95 were identified in the Cyanantheae species, with 13–21 genes located in the IR regions. 99 protein-coding genes were found in the *Cyphia crenata*, with 21 genes in the IR regions. Four conserved rRNAs were checked in every species. The *T*. *caeruleum* plastome encodes 44 types of tRNAs, whereas other species encodes 36–38 (Table 1).

The plastid genome structure comparison using MAUVE software revealed that the plastomes of all the accessions were not conserved, and many rearrangements of gene organization had occurred (S2 Appendix). We identified some obvious differences, such as gene composition, gene order, GC content, IR junction in the plastomes of the Campanulaceae, although the plastid genomes of land plants are commonly supposed to be highly conserved [55].

On the other side, we divided the eleven species within Campanulaceae into four groups according to the phylogenetic results of this study, they were the groups of all species, the Campanuleae, the Cyanantheae and the out-group of *Lobelia erinus* and *Cyphia crenata*. The nucleotide diversity (pi) value of four groups was calculated to evaluate the sequence divergence among the 76 protein-coding genes of plastomes (Fig 2 and S1 Table), with the mean value of 0.06649 in the out-group, 0.05687 in the Campanuleae species, and 0.03394 in the Cyanantheae species. The analysis revealed that all four groups exhibited the high levels of divergence in the *ccsA* and *ndhF* gene of the SSC regions, which indicated that the SSC region might be undergoing rapid nucleotide substitution in species of family Campanulaceae and contain variable information for species authentication and phylogenetic analysis. *Ycf1* and *ycf2* gene were the hotpot regions for each group. Furthermore, we also identified two hotpot regions (*rpl22* and *rps3* gene, pi>0.1) for the group of all species, while the other three groups did not show the high divergence in above two genes. Many fragments of coding genes, such as *atpB*, *matK*, *ndhF*, have been used for phylogenetic reconstructions at various taxonomic levels [56–58]. We could use the hotpot regions acquired from this study to develop the potential markers, which would be helpful not only in identifying the species, but also in the reconstruction of phylogeny within differernt groups of Campanulaceae in further studies.

### IR contraction and expansion

It is well known that the IR regions facilitated the stability of the other regions of the genome by intramolecular recombination, thus limiting recombination between the LSC and SSC regions [59, 60]. The expansion and contraction of IR regions at the borders are considered the major reasons for genome size differences, and are best to study the phylogeny and the plastid genome evolution history of plants [61–63]. We checked the differences of the borders among the IR, LSC and SSC regions of 9 genera. The differences of genes located in the IR region were also examined. Detailed comparisons of the boundaries among the studied plastomes were presented in Fig 3. The *ndhE* gene crossed the IRa and SSC regions for the Campanulaceae species, and the boundary between SSC and IRb regions was in the *ndhF-ndhG* spacer (Fig 3). The *ndhF* gene was complete in the SSC region, more than 200bp away from the IRb region.

Cyanantheae species and *Lobelia erinus* had the same IRa/LSC borders: the *rps19* gene in the LSC region and the *rpl2* gene in the IRa region. The IR regions contained the *rpl2*, *trnI-CAU*, *ycf2*, *trnL-CAA*, *ndhB*, *rps7*, *ycf1*, *rps15*, *ndhH*, *ndhA*, *ndhI*, *ndhG*, and part of *ndhE* genes. It was worth mentioning that *P*. *grandiflorus* had the IRa/LSC boundary spanning the *rpl36* gene. Besides, this species had the similar gene contents to the other species of Cyanantheae, coupled with the *rps8*, *rpl14*, *rpl16*, *rps3*, *rpl22*, *rps19*, and part of *rpl36* gene. The species of this group showed no IR expansion and contraction, which were canonical IR and similar to *L*. *erinus*.

For the Campanuleae species, the IRa/LSC boundary was located between the *trnL-CAA* gene and the *ycf2* gene. There were only eight complete genes, *trnL-CAA*, *clpP* genes, etc., in the IR region. The *ycf2* genes appeared in the LSC regions. The length of the IRa regions of three Campanuleae species, varying in the range of 27,276–29,637 bp, was shorter than the eight other species, which varied from 37,290–45,915 bp (Table 1). Species of Campanuleae occurred the IR-contracted out of LSC, and the large IR contractions have been rarely reported, and the most plausible explanation is considered as illegitimate recombination [64–66].

Plastome of *Cyphia crenata* experienced IR-expaned into LSC, which lead to the largest plastome of studied Campanulaceae (Fig 3). The *petB* gene of *Cyphia crenata* crossed the IRa/LSC region, with 187bp located in the LSC region and 2,595 bp in the IRa region. IR region of *Cyphia crenata* had the part of *petB* gene, *petD* gene, ORF 159, ORF 180 and ORF119, which did not show in the IR regions of the other studied species. We also calculated the ORFs >300 bp in the IRa region, among the eleven species, and the results illustrated that there were five ORFs appearing in the IRa regions of *Cyphia crenata*, with total length of 2,211bp. However, other species had 1–3 ORFs, with length of 324 to 1,230bp (S2 Table). *Cyphia crenata* was the only species indicating the IR region expanded into LSC region. It was hypothesized that the longer sequences of ORFs appearing in the IRa regions might be closely associated with IR expansion. Additionally, the IR region of *Cyphia crenata* had more tandem and dispersed repeats compared with the LSC region and SSC region. Previous studies have suggested that the intramolecular recombination, the occurance of many various repeat sequences, and the insertion-deletions may interpretate the variety of the IR boundary region sequences [59, 67–69], which could also be applied to explain the large IR expansion of *Cyphia crenata*.

The IR expansion and contraction of this study provided new evidence for the classification of Campanulaceae *s*.*str*. at the genome level. Based on the species included in this study, the group of Cyanantheae species with canonical IR was sister to the Campanuleae species having the IR-contracted out of LSC regions, which was consistent with previous studies about the subdivision of Campanulaceae *s*.*str*. [4, 10, 11]. In addition, the IRa/LSC boundary and the IR contents of Cyanantheae species were similar but different from Campanuleae species, with the exception of *Platycodon grandiflorum*.

Overall, the junction positions of LSC/IRa regions varied slightly in the plastid genomes of Campanulaceae, and the genes existed in the IRa region were also different in the studied groups. Whereas, the boundary of IRa/SSC of all species had the similar pattern. The events of IR expansion and contraction are helpful to research subdivision of Campanulaceae *s*.*str*. and the genome evolution among the Campanulaceae species.

### Codon usage bias

**Fig 4:**
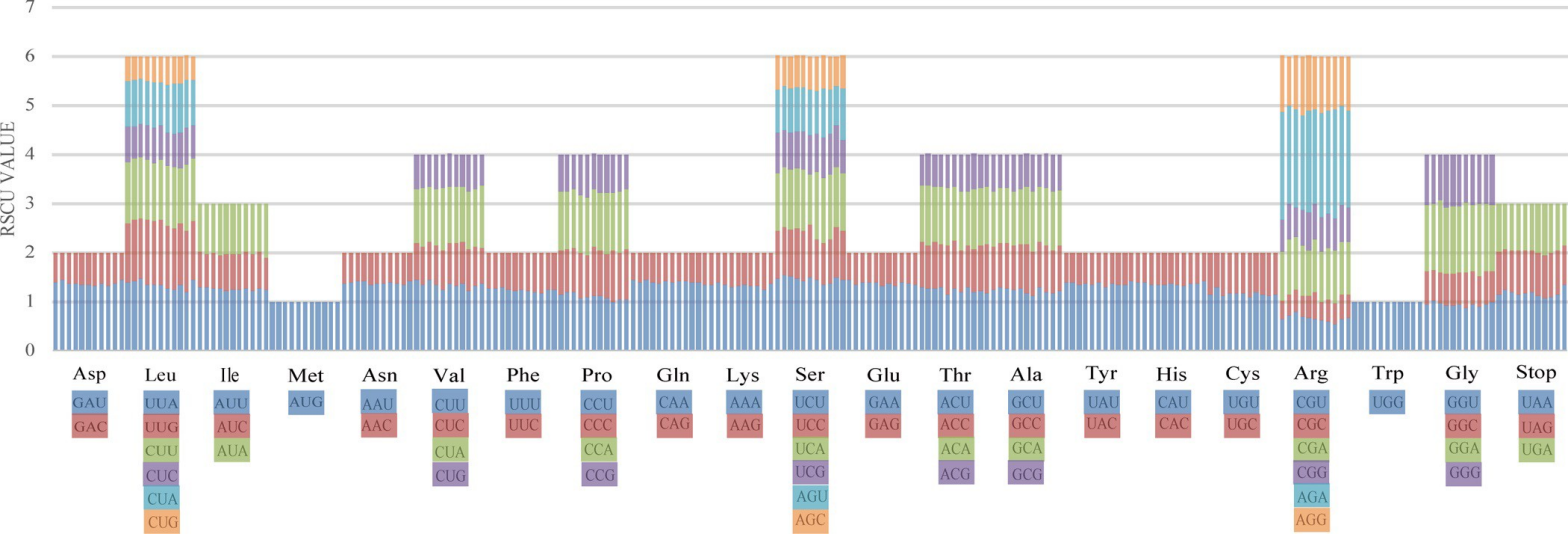
Codon contents of 20 amino acids and stop codons in all protein-coding genes of the Campanulaceae plastomes. The color of the histogram corresponds to the color of codons.

**Fig 5:**
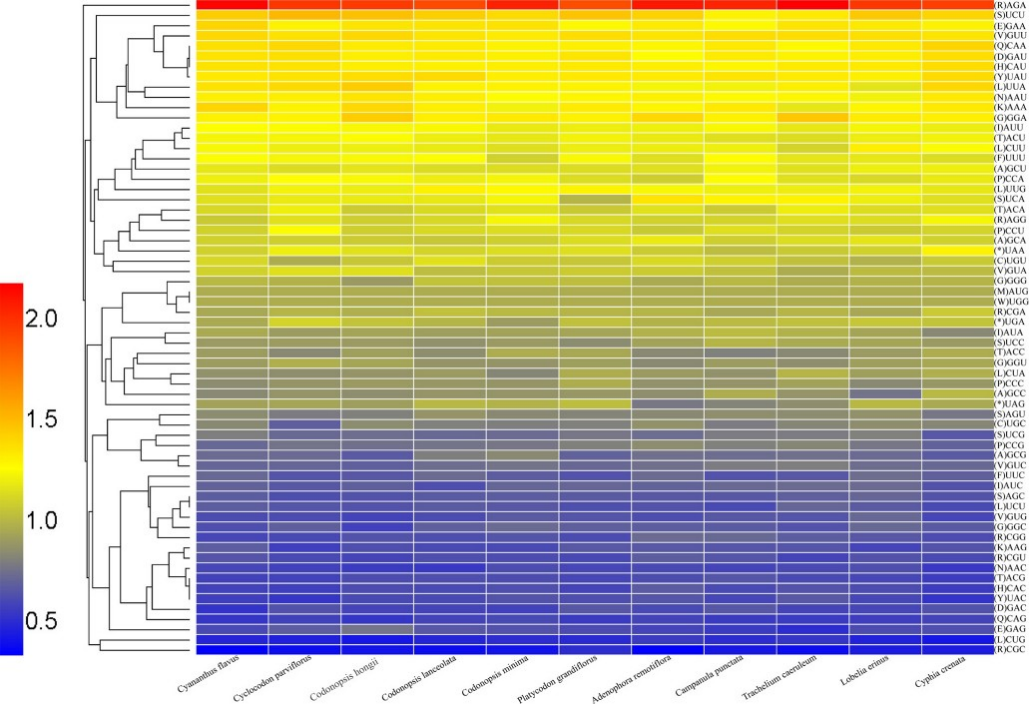
Heatmap analysis for codon distribution of all protein-coding genes of all considered species. Colour key: Higher red values indicate higher RSCU values, and lower blue values indicate lower RSCU values.

The plastid genome of Campanulaceae was detected for its codon usage frequency according to sequences of protein-coding genes and relative synonymous codon usage (RSCU). RSCU refers to the relative probability of a codon encoding a corresponding amino acid synonymous codon, which eliminates the effect of amino acid composition on codon usage [70]. The pattern of the codon preference has the vital role in studying species evolution [71–73]. The analytic varieties provided by statistical analyses of all 76 protein-coding cpDNA and amino acid sequences demonstrated obvious codon preferences. It showed the similarity of protein codons in the Campanulaceae species, of which AGA had the highest frequencies, and CGC had the least occurrence frequencies (Figs 4 and 5). 64 codon preferences were identified, with 20 amino acids and one stop codon involved. The standard ATG codon was typically the start codon for nearly all protein-coding genes. All three stop codons were present, with UAA being the most frequent stop codon in all eleven plastomes. RSCU values of methionine (AUG) and tryptophan (UGG) were equal to one and encoded by only one codon, indicating no codon bias for these two amino acids. All the protein-coding genes were composed of 42,552–48,095 codons as shown in S3 Table.

As shown in Fig 5, the result of the distributions and the visualization of codon usage in the form of heatmaps of Campanulaceae species showed that approximately half of the codons were not frequently used. These codons had the RSCU value of >1, and most of these (25/28, 89.3%) ended with base A or U, resulting in the bias for A/T bases. About half of codons had the RSCU value of <1, and most of those (27/34, 79.41%) ended with base C or G. The third codon shows a high A/U preference, which is a common phenomenon in plastid genomes of higher plant [74–76]. The high RSCU value is possibly caused by the function of the amino acid or the structure of the peptide to avoid mistakes in transcription [77].

### Analysis of repeats

**Fig 6:**
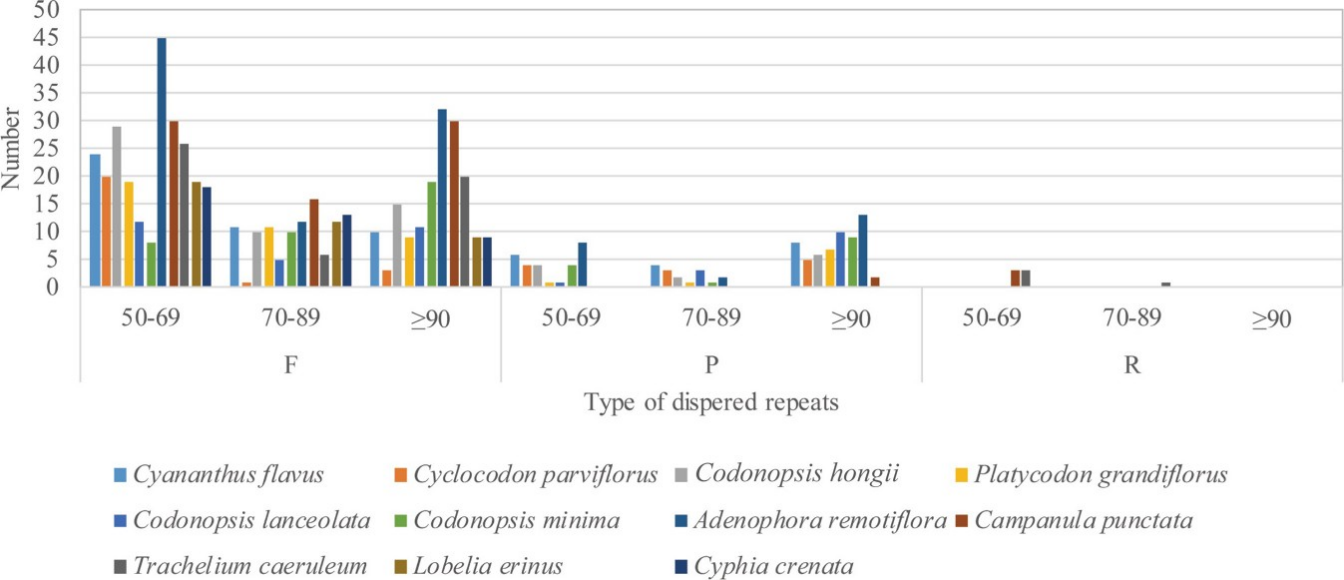
Frequency of three types of dispersed repeats by length. (F: forward, P: palindrome, R: reverse).

This analysis of repeats was only token one IR into account. In the majority of the studied species, the most dispersed repeats were forward, then palindromic, and the least reverse. The comparison analyses (Fig 6) revealed that most of the forward repeats were 50–69 bp, and the longest repeats with length of 1,009 bp, were detected in the *T*. *caeruleum*, followed by *Campanula punctata* of 640 bp length, and *Adenophora remotiflora* of 620 bp length, which were much longer than other species studied. Besides, in the group of Campanuleae species, dispersed repeats were mainly distributed in non-coding regions (IGS) (S4 Table). Long repeat sequences may be useful to do phylogenetic analysis and increase plastid genome rearrangements [73, 78, 79].

The results also displayed that among the tandem repeats, the repeats located in the spacer of *rpl2-trnI CAU* had appeared in the clade of Cyanantheae and the out-group, but not shown in the Campanuleae species (S5 Table) which had the IR contraction and did not show the *ycf2* gene in the IR region. It indicated that the lack of repeats in *rpl2-trnI CAU* might be linked to the IR contraction. Most and variable tandem repeats (except for species of *Codonopsis minima*, *Trachelium caeruleum* and *Cyphia crenata*) were located in the CDS regions, which might accelerate evolution of coding and regulatory sequences [80].

**Fig 7:**
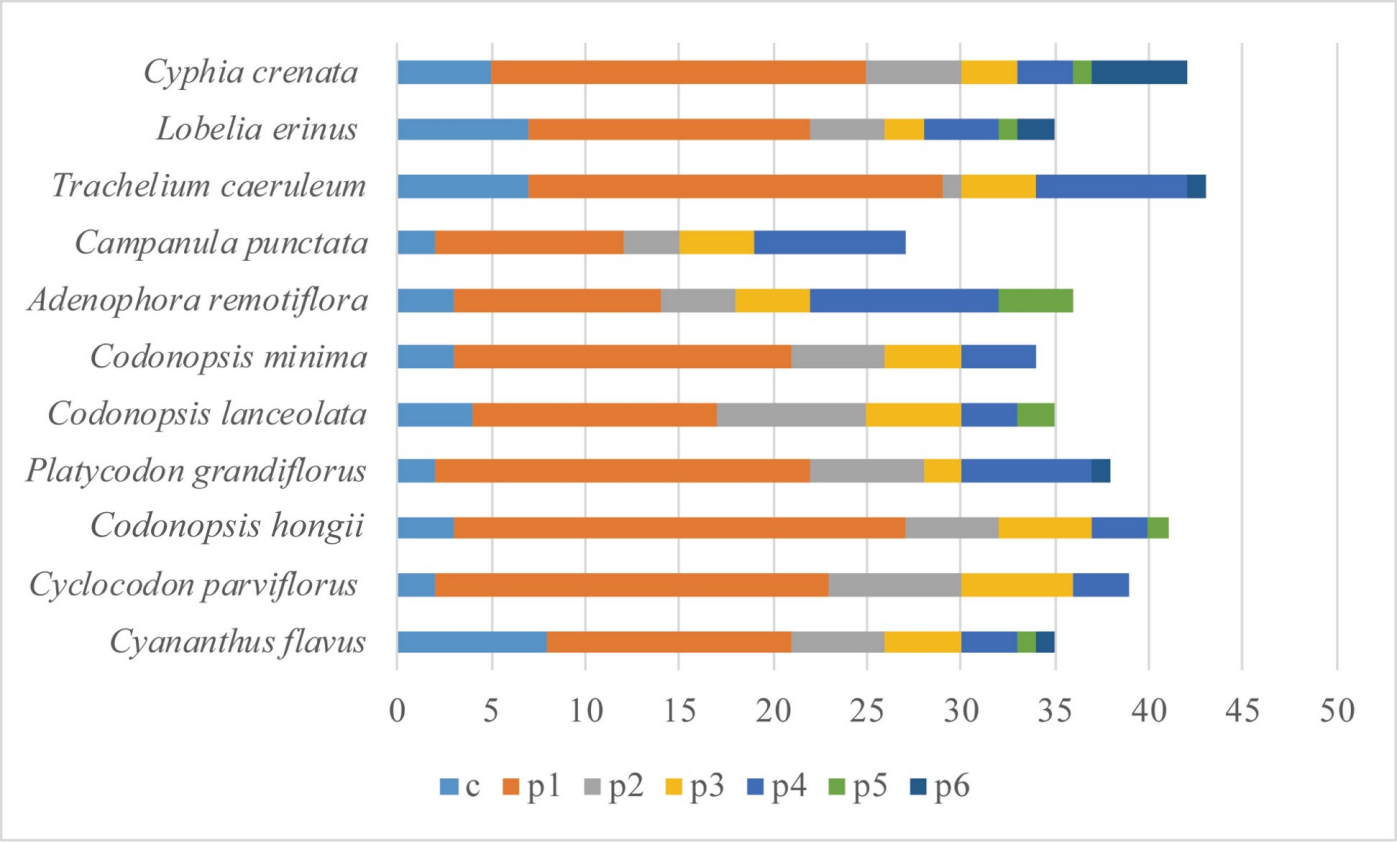
The distribution maps of simple sequence repeats (SSR). Classification of SSRs by repeat types. p1, mononucleotides (mono-); p2, dinucleotides (di-); p3, trinucleotides (tri-); p4, tetranucleotides (tetra-); p5, pentanucleotides (penta-); p6, hexanucleotides (hex-); c, compound.

A large proportion of SSRs was found in the non-coding regions (IGS). We identified A/T/G mononucleotide repeats (p1), while the majority of the dinucleotide repeat sequences (p2) were comprised of AT/TA repeats, and the TG, CA, AC and GT repeats were also found. Furthermore, A and T were the most frequent bases in all SSR types, which resulted in the bias for the studied plastomes. About half of the species had the compound repeats (S6 Table and Fig 7). Most simple sequence repeats (SSRs) are widely used for species authentication, phylogenetic analysis, and population genetics because of their high levels of polymorphism [81–84]. Microsatellites have a great influence on the genome recombination and rearrangement by their wide distribution across the entire genome [85–87]. The other types of mono-, di-, tri-, tetra- and penta- nucleotide were identified at a much lower frequency among the Campanulaceae species and other plants [88–90].

**Fig 8:**
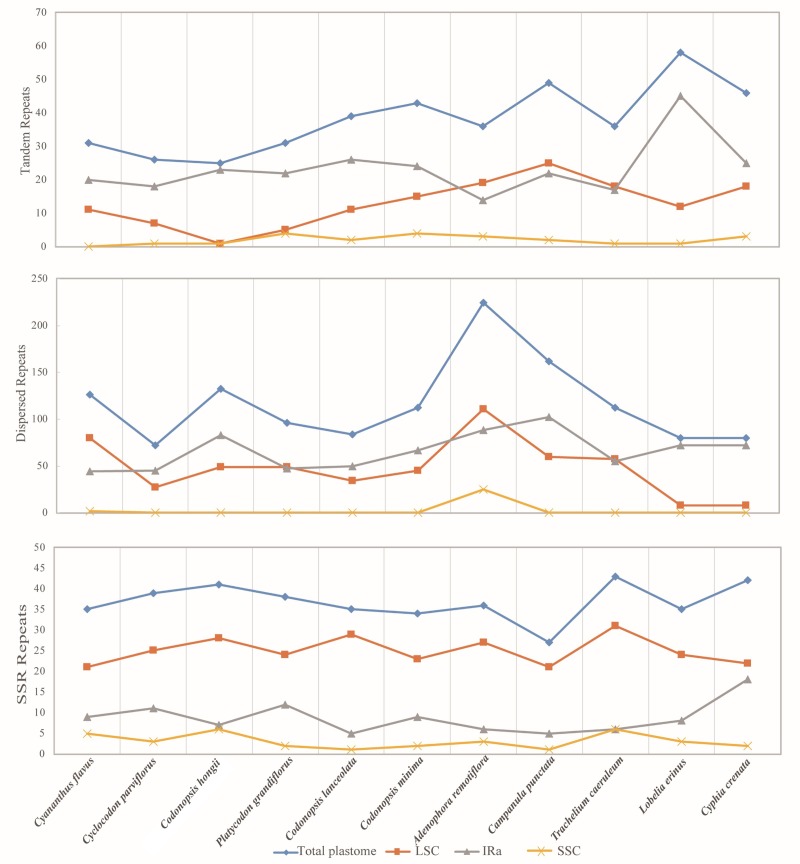
Repeat number in the different regions of Campanulaceae plastomes, including Tandem repeats, Dispersed repeats and SSR repeats. The yellow line refers to SSC regions, the gray lines refers to IRa regions, the orange line refers to LSC regions, and the blue line refers to the total plastome.

The total plastome regions of all Campanulaceae possessed the highest number of tandem, dispersed and SSR repeats (S4–S6 Tables and Fig 8), and SSC regions had the lowest number of these repeats. SSR repeats of LSC regions contained higher number of repeats compared with IRa and SSC regions. Tandem repeats of IRa regions had more repeats than LSC and SSC regions in some species, while less in species of Campanuleae. However, Campanuleae had more tandem repeats in LSC regions, which may be guessed that this phenomena is relevant to the IR contraction [91–93].

There was nearly no dispersed repeats in the SSC regions, except the *Adenophora remotiflora* with more than 89 dispersed repeats. The results showed that dispersed repeats of IR regions of Cyanantheae appeared more frequently than in LSC regions, except for *Cyananthus flavus*. The presence of all types of repeats demonstrated that the locus was a crucial hospot for genome reconfiguration [94–97]. Moreover, the repeats of plastid genomes will be helpful for identifying polymorphisms at the species level for deducing distant phylogenetic relationships among Campanulaceae species. Repeats were previously inferred to associate with plastome structural variation [98–101]. In this study, the plastomes of all studied species possessed large amount of repeats and longer repeats, and presented the structural variations. These together supposed that repeats might also affect size variation in the Campanulaceae plastomes.

### Positive selection analysis

We compared the ratio of non-synonymous (dN) and synonymous (dS) substitution for 76 protein-coding genes among the newly sequenced species with other eight species. We focused on the Bayes Empirical Bayes (BEB) analysis of Paml and the highly positively selected sites of P>99% (**) because one slightly positive selection had more than 10 positive sites of P>95%. Finally, fourteen genes with highly positively selected sites within the Campanulaceae family were identified (S7 and S8 Tables). Those genes contained one subunit of Protease (*clpP*), two NADH-dehydrogenase subunit genes (*ndhD*, *ndhI*), two photosystem II subunit genes (*psbL*, *psbN*), one ribosome large subunit gene (*rpl16*), six ribosome small subunit genes (*rps3*, *rps4*, *rps8*, *rps11*, *rps12*, *rps18*), and the *ycf1*, *ycf2* gene. According to the M2 and M8 models, *ndhI*, *psbI* and *rps3* only had one sites under highly positive selection. The gene *ycf1* and *ycf2* harbored more than 30 highly positive selections, followed by *clpP* (7,11), *ndhD* (10, 0), *psbN* (0,2), *rpl16* (3,4), *rps4* (3,6), *rps8* (0,2), *rps11* (1,1), *rps12* (18, 22). Likelihood ratio tests (M0 vs. M3, M1 vs. M2 and M7 vs. M8) supported the presence of highly positively selected codon sites (S8 Table). Some studies have indicated that *ycf1* is required for plant viability and encodes Tic214, which is a vital component of the TIC complex in *Arabidopsis* [102–104]. Most genes under positive selection have the functions in genetic system or photosynthesis, which demonstrate that the functional genes of plastid have important significance during the plant evolution [105–108].

There existed limitation in the study of natural selection by using branch and site modes separately because for the majority of genes in a specified branch, only few sites were under positive selection, however, branch-site model allowed us to detect the various selective pressure on the labeled foreground lineage against the remanent background branches [48]. After the analysis of BEB, we found 96 sites under potentially positive selection in the 76 protein-coding genes with posterior probabilities more than 0.95 and 10 sites greater than 0.99 (S9 Table). The branches of Cyanantheae, Campanuleae and out-group all showed there were positively selected sites in *ycf1* and *ycf2*, and there were more detected on the branch of Cyanantheae for *ycf2*. Campanuleae lineage demonstrated the positively selected sites in *rpl16* but did not reveal the positively selected sites in *rps2*, *rps3*, *rps4*, *rps11* and *rps15* although the LRT *p*-value was less than 0.05. The out-group branch showed one positively site in *ndhI*. *rpoA* gene also did not have positively selected sites in the branch of Cyanantheae. It has been shown that the high rate of molecular evolution existing in numberous genes following genome duplication actuates the functional changes [109, 110]. Besides, the positive selection is concerned with the shift of function and environment [109, 111]. Therefore, positively selected sites detected in this study may drive the protein-coding genes allowing occupation of diverse habitats [48, 109].

### Phylogenetic analysis

In recent years, more plastid genome database provides an important basis for the determination of the evolutionary, taxonomic, and phylogenetic studies of plants [51, 112–116].

**Fig 9:**
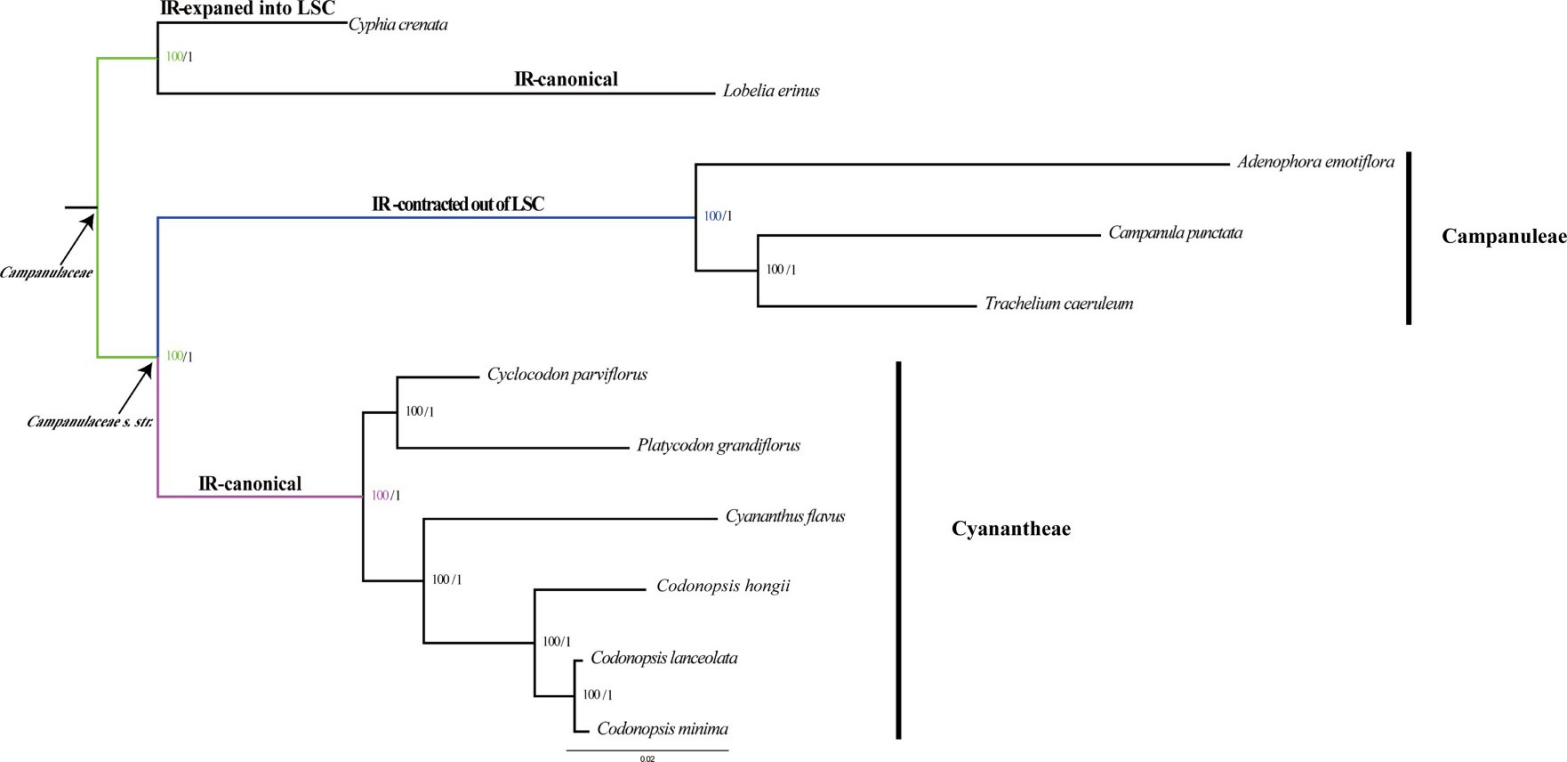
Phylogenetic relationship of all Campanulaceae species by using the 76 protein-coding genes, based on the Maximum likelihood (ML) analysis and Bayesian inference (BI) analysis.

Phylogenetic analysis was performed by ML and BI nucleic acid analyses based on the 76 aligned sequences of plastomes (Fig 9). *Lobelia erinus* and *Cyphia crenata* were used as out-group. The two typologies showed similar phylogenetic patterns. The ML tree revealed that Campanulaceae *s*.*str*. formed a monophyletic clade, and Cyanantheae and Campanuleae were also monophyletic. The bootstrap value of previous researches on the phylogenetic relationships of Cyanantheae was relatively low by using ITS sequence and several plastid markers [4,11]. However, the relationships of Cyanantheae species were well supported in this study. All nodes in the phylogenetic tree were strongly supported, with 100% bootstrap (BP) values and 1.00 Bayesian posterior probabilities (PP). From phylogenetic analysis, Cyanantheae species were divided into two clades. One clade consisting of *Cyclocodon parviflorus* and *P*. *grandiflorus* was the earlier diverging lineage in the group of Cyanantheae. The other clade was composed of *Cyananthus flavus*, *Codonopsis hongii*, *Codonopsis lanceolate* and *Codonopsis minima*. *Codonopsis hongii* was a sister species to other *Codonopsis* species. *Cyclocodon parviflorus* had a close relationship with *Platycodon grandiflorus*. Previous studies had demonstrated that *Cyclocodon* was restored as the separate genus only based on the morphology of pollen and seed coat, plus the gross morphological characters [12, 117]. In this study, *Cyclocodon* was not closely related to *Codonopsis* and had different structures of plastid genomes compared with *Codonopsis* species (Fig 3), which supply the extra evidence for confirming *Cyclocodon* at the generic rank. *Cyananthus* were treated as the generic rank by the former researches, but the phylogenetic relationships between *Codonopsis* and *Cyananthus* were weakly supported [12]. Nevertheless, *Cyananthus flavus* being related to all studied *Codonopsis* species was demonstrated in our study with strong supports based on the 76 protein coding genes. Therefore, successful phylogenetic construction for eleven Campanulaceae species studied here imply that plastid genome database will be a potentially useful resource for molecular phylogeny studies within the order Cyanantheae.

The results also indicated that it was helpful to illustrate phylogenetic analysis of species in the family Campanulaceae. The phylogenetic tree constructed in this study showed that Cyanantheae formed a sister clade to Campanuleae clade (Fig 9), which is consistent with the previous studies [9, 11]. Therefore, it is hypothesized that Cyanantheae had an earlier divergence among the Campanulaceae from a common ancestor than Campanuleae species because Campanuleae had a unique IR contraction structure (Fig 3). The phylogenetic relationships of Campanuleae have been explored by using the coding regions of plastomes [24].

## Conclusions

We first reported the complete plastid genome sequences of three Asian Cyanantheae species (*Cyananthus flavus*, *Cyclocodon parviflorus*, and *Codonopsis hongii*) and compared these to published species in the family Campanulaceae. The results of the genome structural comparison indicated the large amount of rearrangements and various repeat sequences. The junctions between the LSC region and IRa region manifested the diverse locations in different clades. IR contraction/expansion might be explained by the multiple repeat sequences, the indels and the recombination. Fourteen genes with highly positively selected sites within the Campanulaceae family had been identified, and most of them were genetic system or photosynthesis related genes. Branch-site model revealed many positively selected sites in certain genes on the specified branches, which may offer the important significaces for the adaptive evolution. We also discussed the type of the codon preference, which had the vital roles in studying species’ evolution. Six coding-regions (*ccsA*, *ndhF*, *rpl22*, *rps3*, *ycf1* and *ycf2*) in the highly variable regions will be utilized as potential molecular markers for constructing the phylogenetic relationships of the family Campanulaceae. Phylogenetic analysis indicated that *Cyananthus* was more closely related to *Codonopsis* compared with *Cyclocodon* and clearly showed the relationship among the Cyanantheae species. The plastid genomes will contribute to the development of genetic resources in resolving the phylogenetic analysis and species authentication of Campanulaceae, and in facilitating the exploration their structural differences. Nevertheless, only limited species were shown in this study, and thus, we believe that further studies that include various species having the information of plastomes, are needed to clarify the molecular evolution and phylogenetic relationships of Campanulaceae.

## Supporting information

S1 Table The midpoint and pi value among the different groups of Campanulaceae species. (XLSX)

S2 Table ORFs (more than 300bp) showing in the plastomes. (XLSX)

S3 Table Putative preferred codons in the Campanulaceae plastid genomes. (XLSX)

S4 Table Dispersed repeats found in the plastomes. (XLSX)

S5 Table Tandem repeats among the studied species. (XLSX)

S6 Table SSRs showing in the plastomes of Campanulaceae species. (XLSX)

S7 Table Maximum likelihood parameter estimates for the 76 genes of Campanulaceae species. (XLSX)

S8 Table Likelihood ratio test (LRT) of the variable ω ratio using site model. (XLSX)

S9 Table Parameter estimates and likelihood values for 76 protein-coding genes inferred using branch-site model. (XLSX)

S1 Appendix Primers used for assembly validation. (DOCX)

S2 Appendix Mauve result of the plastid genomes of eleven Campanulaceae species. (DOCX)
