# Supplementary material for: Comparative analysis of plastid genomes within the Campanulaceae and phylogenetic implications
Source: PLoS One. 2020 May 14;15(5):e0233167. doi: 10.1371/journal.pone.0233167 (PMC7224561; doi:10.1371/journal.pone.0233167)
Supplement: S1 Appendix — (DOCX) [file pone.0233167.s010.docx]

**S1 Appendix. Primers used for assembly validation.**

| **Primer** | **Sequence(5'>3')** | **Product size (bp)** |
| --- | --- | --- |
| **LSC/IRa** | AGTCGGTGCAAATTCTCCCA GTGGGGAATCTTGGGGTGAG | 540 |
| **IRa/SSC** | TGCTCGAACATGTACTTGTTTTGA CTTTCTGGGCGAGGGTATCA | 506 |
| **SSC/IRb** | CACATCTGTGGGACAGGCTC AGGGCTCTTATGTGTCTTGAACT | 554 |
| **IRb/LSC** | GCTAGGTAAGCGCCCTGTAG AAGAGGGCGTTATTGGTCCC | 503 |

DNA amplification was performed for an initial 180s at 94 °C, followed by 35 cycles of 60s at 94 °C, 120s at 50 °C, 60s at 72 °C and a final 5 min at 72 °C. Reactions were carried out in a volume containing 2.0 mm/L MgCl_2_, 0.6 mm/L dNTP, 1μL of 10× buffer, 0.3 μmol/L of each primer, 1 U Taq DNA and 30 ng DNA template.
