# Supplementary figures and images for: Comparative analysis of plastid genomes within the Campanulaceae and phylogenetic implications
Source: PLoS One. 2020 May 14;15(5):e0233167. doi: 10.1371/journal.pone.0233167 (PMC7224561; doi:10.1371/journal.pone.0233167)

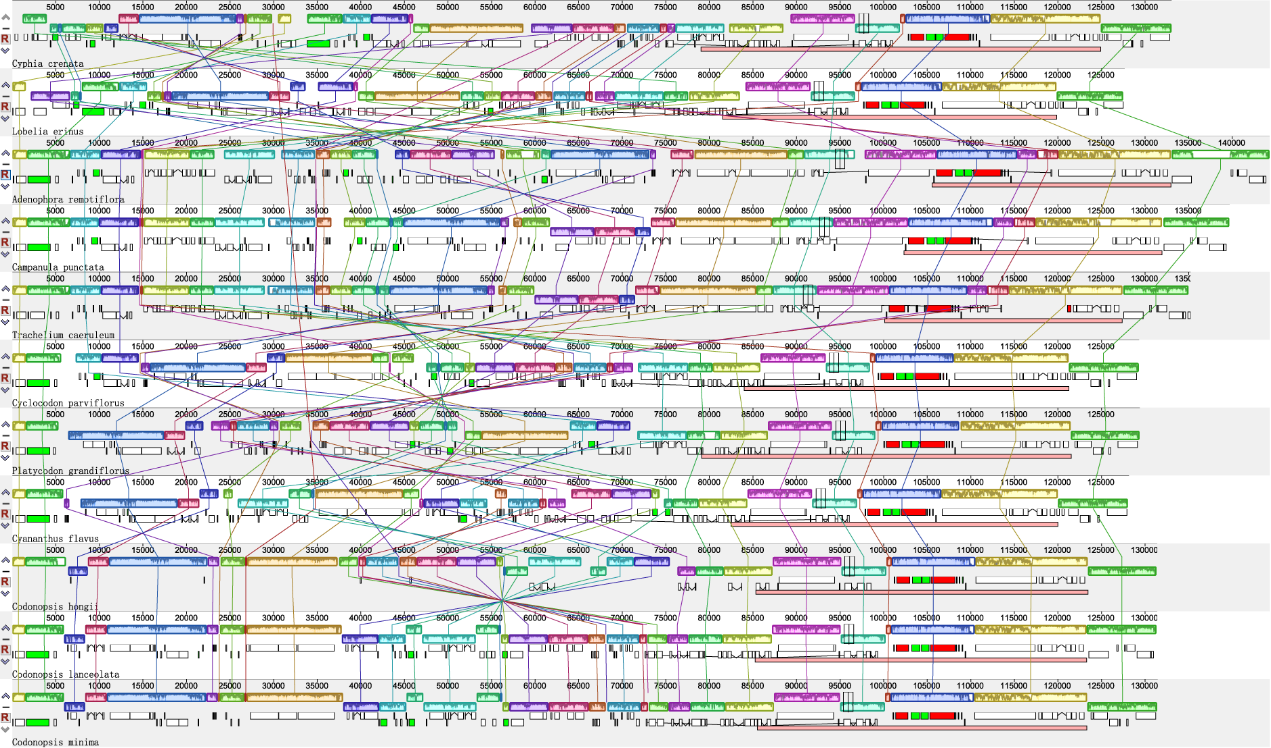


**S2 Appendix. Mauve result of the plastid genomes of eleven Campanulaceae species.**

Supplement: S2 Appendix — (DOCX) [file pone.0233167.s011.docx]
